# Circular RNAs and RNase L in PKR activation and virus infection

**DOI:** 10.1186/s13578-019-0307-x

**Published:** 2019-05-27

**Authors:** Zhi-Ming Zheng

**Affiliations:** Tumor Virus RNA Biology Section, RNA Biology Laboratory, Center for Cancer Research, National Cancer Institute, NIH, Frederick, MD USA

**Keywords:** Circular RNA, PKR, Innate immunity, RNase L, Systemic lupus erythematosus

## Abstract

Circular RNAs (circRNAs) from back-splicing have been found in every cell and tissue. By binding to miRNAs and proteins or even by encoding small proteins, circRNAs are now emerging as important regulators in modulating transcription, RNA splicing and interference. The highlighted discovery reports an important role of circRNAs in antiviral innate immunity by binding PKR as PKR inhibitors. Rapid degradation of circRNAs by activated RNase L from virus infection or poly I:C stimulation is required to free PKR for its activation. Systemic lupus erythematosus patients remark with circRNA reduction and aberrant PKR activation.

Intracellular foreign RNAs are recognized by two groups of intracellular RNA sensors. Immune sensing receptors recognize 5′-triphosphate (ppp) ssRNA and short dsRNA to induce immune responses via activation of transcription and production of cytokines. This group includes RIG-I (retinoic acid-inducible gene I), MDA-5 (melanoma-differentiated gene 5) and TLRs (Toll-like receptors). Nucleic acid receptors directly recognize and act on dsRNAs in different size to execute antiviral activities by blocking translation and inducing degradation and modification of pathogenic dsRNA. The latter group includes PKR (dsRNA-activated protein kinase R), OAS (oligoadenylate synthetase), and ADAR1 (adenosine deaminase acting on RNA 1).

A viral RNA genome in a circular form was initially recognized in viroids in 1976 and in human hepatitis delta virus in the early 1980s. A large number of circular RNAs (circRNAs) produced from primary mRNA (pre-mRNA) splicing in eukaryotic cells were also subsequently noticed to be the splicing intermediate intronic lariats [1] and exon back-splicing products [2]. Although the circular lariats are commonly produced by splicing of each pre-mRNA intron [3] and subject to digestion by a debranching enzyme DBR1 (debranching RNA lariats 1), the back-splicing derived circRNAs initially recognized in 1996 [4] are considerably in low production efficiency (< 1% of canonical splicing) and the functional potential of the back-splicing derived circRNAs remains elusive. Due to their circular form in nature, circRNAs are relatively stable and resistant to linear RNA decay machineries in eukaryotic cells. In a recent article published online by Cell, April 25, 2019, Chu-Xiao Liu and Ling-Ling Chen et al. from Shanghai Institute of Biochemistry and Cell Biology, Chinese Academy of Sciences, reported their astonishing discoveries on structure and degradation of the back splicing-derived circRNAs in regulation of PKR activation in innate immunity [5]. In this report, the authors found that stimulation of HeLa cells with poly I:C, being widely used to mimic pathogenic dsRNAs, or viral infection with an RNA virus, encephalomyocarditis virus (EMCV), led to dramatic and rapid reduction of all examined circRNAs with a turnover half-life of ~ 1 h. The observed fast turnover of circRNAs upon poly I:C treatment was not due to the transcriptional level interference, but specific to poly I:C and EMCV. The catalytic activity of an endoribonuclease RNase L was essential for circRNA degradation. It has been known for long time that pathogenic dsRNA binds to and activates OAS to produce 2′,5′-linked oligoadenylates (2-5A) which then activate cytoplasmic RNase L to catalyze the degradation of viral and host RNAs to restrict virus infection. By knockout (KO) of RNase L expression in HeLa cells, the authors discovered that the activated RNase L is the key enzyme for circRNA degradation upon poly I:C stimulation or EMCV infection.

Further investigation to identify the circRNA-associated proteins revealed that the circRNAs, both circPOLR2A and circCAMSAP1, preferentially bind to nucleic acid receptors PKR, NF90 (ILF3 isoform-2) and OAS, whereas their linear RNA forms bind to immune-sensing receptors TLR3, RIG-I and MDA-5. Interestingly, this binding of circPOLR2A to PKR could be blocked by a short 33-bp dsRNA of which binding doesn’t activate PKR, but the same short RNA in linear form had no effect. These observations led the investigators looking into the question whether the circRNAs could form intramolecular RNA duplexes to bind and activate PKR. Surprisingly, they discovered that each HeLa cell may contain ~ 9000–10,000 copies of circRNAs and each circRNA bears at least 1–4 intra-dsRNA regions in size of 16–26 bps, leading the authors to hypothesize that the short dsRNA region in a circRNA binds PKR in normal cell condition, but not activates PKR because of its short size and thus functions as a PKR suppressor. Further experimental approaches by ectopic expression of circRNAs or by stimulation of RNase L KO cells with poly I:C confirmed this important function of circRNAs in suppression of PKR activation and in innate immunity against EMCV infection.

More strikingly, the authors in this report [5] discovered a higher level of the activated PKR, some spontaneously activated RNase L, and reduced level of circRNAs in the peripheral blood mononuclear cells isolated from systemic lupus erythematosus (SLE) patients over that from the normal control individuals. Ectopic expression of circPOLR2A in T cells isolated from SLE patients could block PKR phosphorylation and PKR activation. SLE is an autoimmune disease characterized by production of numerous auto-antibodies and type I IFN [6]. These important observations in SLE patients provide the strong evidence that imbalanced production and function of the identified circRNAs might be related to the pathogenesis of autoimmune diseases.

Together with the findings of circRNA binding PKR and sensitive to RNase L digestion, the study [5] proposes a paradigm-shift model on how endogenous circRNAs may function to sponge abundant dsRNA-binding PKR in cells to prevent PKR activation by endogenous dsRNAs such as mitochondria-derived mtRNAs and inverted Alu repeats-derived IRAlus [7]. However, the report also raises many questions than answers for future investigation. For example, PKR is primarily a cytoplasmic protein and RNA back-splicing is a nuclear event. Are the intramolecular RNA duplexes in a circRNA folded immediately after back-splicing in the nucleus or later in the cytoplasm? If in the nucleus, these newly formed circRNAs with short dsRNA stems could be bound by highly abundant nuclear NF90 (ILF3 isoform-2) [8] (Fig. 1). How does a NF90-bound circRNA export from the nucleus to the cytoplasm [9] for cytoplasmic PKR interaction? During virus infection or poly I:C stimulation, which dsRNA-binding factor is activated first, RNase L or PKR? According to the proposed model by the authors in the report, degradation of circRNAs by activated RNase L is perhaps a pre-required step to free PKR from the bound circRNAs (Fig. 1). Although to address each of these questions will be a challenging, prospective topic, the reported study opens a new avenue guiding the next wave of discoveries in circRNA research.

**Fig. 1:**
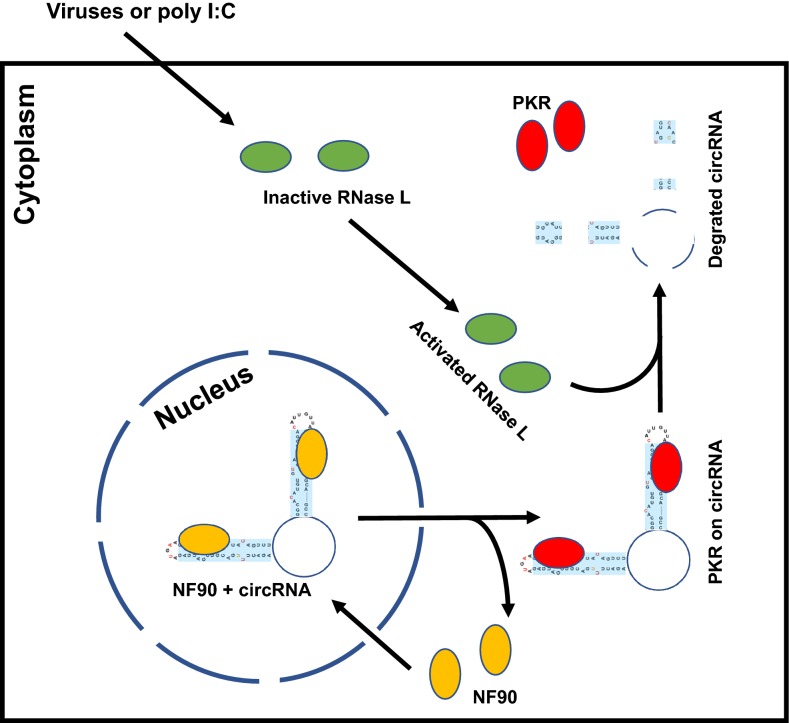
A proposed model depicting a circRNA shuttling with nuclear acid receptors from the nucleus to the cytoplasm to act as a PKR inhibitor. The illustration based on the report [5] shows a newly formed circRNA with two short dsRNA stems in association with nuclear NF90 (ILF3 isoform-2) [8, 9]. After exported with a circRNA to the cytoplasm, NF90 is released from the cytoplasmic circRNA and replaced by cytoplasmic PKR. The free NF90 will either shuttle back to the nucleus or subjects to protein degradation in the cytoplasm. Thus, the cytoplasmic circRNA serves as a PKR sponge and prevents PKR activation. Activated RNase L by RNA virus infection or by poly I:C stimulation binds to the circRNA single-stranded regions, leading to rapid circRNA decay and release of PKR for PKR activation

## Data Availability

Not applicable.

## Abbreviations

circRNA: circular RNA
ssRNA: single-stranded RNA
dsRNA: double-stranded RNA
poly I:C: polyinosinic:polycytidylic acid
PKR: dsRNA-activated protein kinase R
RIG-I: retinoic acid-inducible gene I
MDA-5: melanoma-differentiated gene 5
TLRs: toll-like receptors
OAS: oligoadenylate synthetase
ADAR1: adenosine deaminase acting on RNA 1
EMCV: encephalomyocarditis virus
2-5A: 2′,5′-linked oligoadenylates
NF90: nuclear factor 90
IFN: interferon
SLE: systemic lupus erythematosus
ILF3: interleukin enhancer binding factor 3
