# Correction to: Circular RNAs and RNase L in PKR activation and virus infection

**DOI:** 10.1186/s13578-019-0320-0

**Published:** 2019-07-17

**Authors:** Zhi-Ming Zheng

**Affiliations:** Tumor Virus RNA Biology Section, RNA Biology Laboratory, Center for Cancer Research, National Cancer Institute, NIH, Frederick, MD USA

## Correction to: Cell Biosci (2019) 9: 43 10.1186/s13578-019-0307-x

In the publication of this article [1], there are a few errors in the article.

This has now been included in this correction.

The error in Fig. 1: degrated circRNA.

Should instead read: degraded circRNA.

The corrected Fig. 1 is given here.

**Fig. 1:**
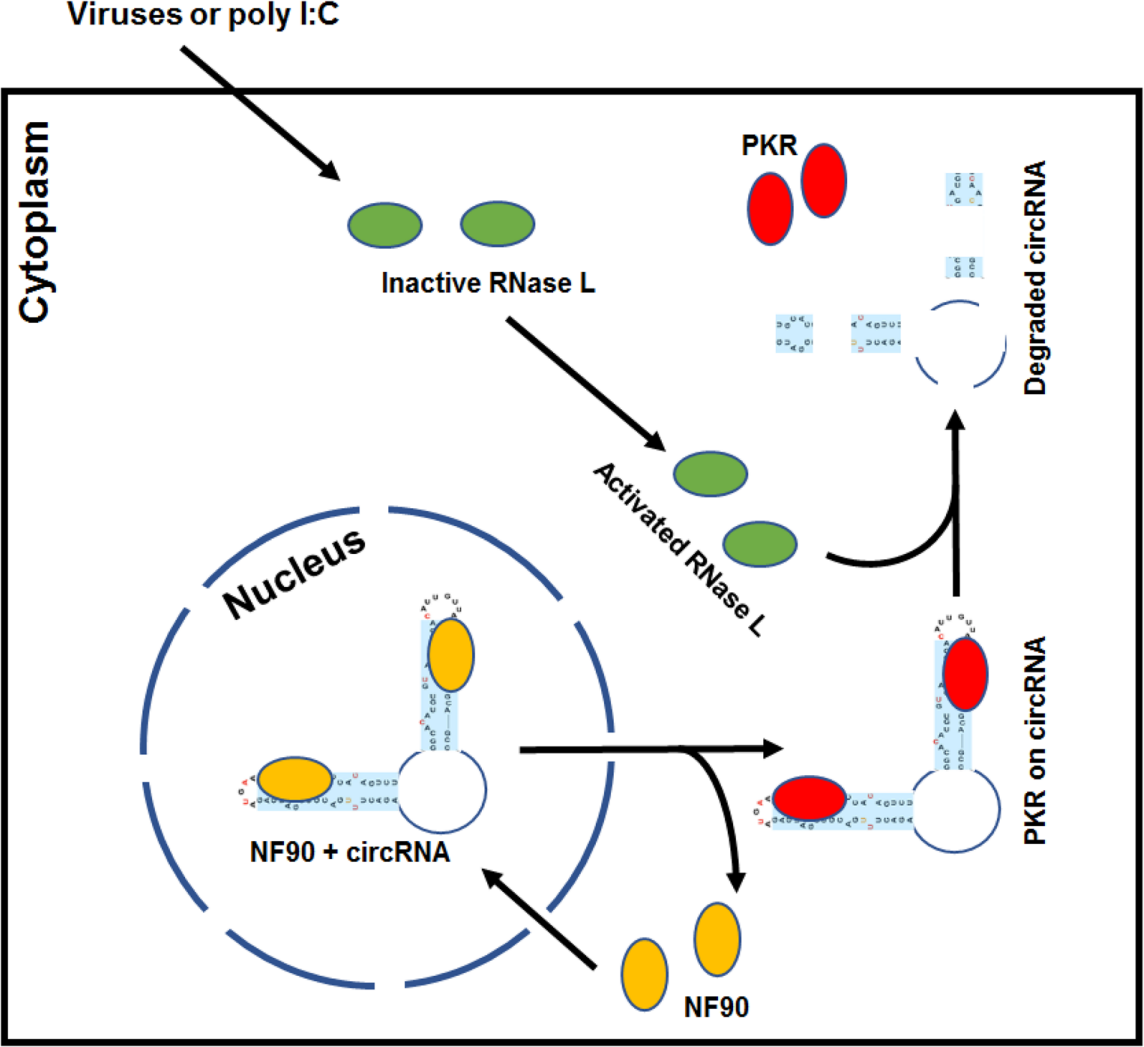
A proposed model depicting a circRNA shuttling with nuclear acid receptors from the nucleus to the cytoplasm to act as a PKR inhibitor. The illustration based on the report [5] shows a newly formed circRNA with two short dsRNA stems in association with nuclear NF90 (ILF3 isoform-2) [8, 9]. After exported with a circRNA to the cytoplasm, NF90 is released from the cytoplasmic circRNA and replaced by cytoplasmic PKR. The free NF90 will either shuttle back to the nucleus or subjects to protein degradation in the cytoplasm. Thus, the cytoplasmic circRNA serves as a PKR sponge and prevents PKR activation. Activated RNase L by RNA virus infection or by poly I:C stimulation binds to the circRNA single-stranded regions, leading to rapid circRNA decay and release of PKR for PKR activation

The error:

Nucleic acid receptors directly recognize and act on dsRNAs in different size to execute antiviral activities by blocking translation and inducing degradation and modification of pathogenic dsRNA.

Should instead read:

Nucleic acid receptors directly recognize and act on dsRNAs of different sizes to execute antiviral activities by blocking translation and inducing degradation and modification of pathogenic dsRNA.

The error:

Although the circular lariats are commonly produced by splicing of each pre-mRNA intron [3] and subject to digestion by a debranching enzyme DBR1 (debranching RNA lariats 1), the back-splicing derived circRNAs initially recognized in 1996 [4] are considerably in low production efficiency (< 1% of canonical splicing) and the functional potential of the back-splicing derived circRNAs remains elusive.

Should instead read:

Although the circular lariats are commonly produced by splicing of each pre-mRNA intron [3] and subject to digestion by a debranching enzyme DBR1 (debranching RNA lariats 1), the back-splicing derived circRNAs initially recognized in 1996 [4] are in considerably low production efficiency (< 1% of canonical splicing) and the functional potential of the back-splicing derived circRNAs remains elusive.

The error:

These observations led the investigators looking into the question whether the circRNAs could form intramolecular RNA duplexes to bind and activate PKR.

Should instead read:

These observations led the investigators to look into the question on whether the circRNAs could form intramolecular RNA duplexes to bind and activate PKR.

The error:

Surprisingly, they discovered that each HeLa cell may contain ~ 9000–10,000 copies of circRNAs and each circRNA bears at least 1–4 intra-dsRNA regions in size of 16–26 bps, leading the authors to hypothesize that the short dsRNA region in a circRNA binds PKR in normal cell condition, but not activates PKR because of its short size and thus functions as a PKR suppressor.

Should instead read:

Surprisingly, they discovered that each HeLa cell may contain ~ 9000–10,000 copies of circRNAs and each circRNA bears at least 1–4 intra-dsRNA regions in size of 16–26 bps, leading the authors to hypothesize that the short dsRNA region in a circRNA binds PKR in normal cell condition, but does not activate PKR because of its short size and thus functions as a PKR suppressor.

The error:

Further experimental approaches by ectopic expression of circRNAs or by stimulation of RNase L KO cells with poly I:C confirmed this important function of circRNAs in suppression of PKR activation and in innate immunity against EMCV infection.

Should instead read:

Further experimental approaches by ectopic expression of circRNAs or by stimulation of RNase L KO cells with poly I:C confirmed this important function of circRNAs in the suppression of PKR activation and in innate immunity against EMCV infection.

The error:

However, the report also raises many questions than answers for future investigation.

Should instead read:

However, the report also raises more questions than answers for future investigation.
